# A chemogenomic approach is required for effective treatment of amyotrophic lateral sclerosis

**DOI:** 10.1002/ctm2.657

**Published:** 2022-01-22

**Authors:** Georgios Pampalakis, Georgios Angelis, Eleni Zingkou, Kostas Vekrellis, Georgia Sotiropoulou

**Affiliations:** ^1^ Department of Pharmacology – Pharmacognosy School of Pharmacy Aristotle University of Thessaloniki Thessaloniki Greece; ^2^ Department of Pharmacy School of Health Sciences University of Patras Rion‐Patras Greece; ^3^ Biomedical Research Foundation Academy of Athens Athens Greece

**Keywords:** amyotrophic lateral sclerosis (ALS), animal models, chemogenomics, disease heterogeneity, small molecules, stratification

## Abstract

ALS is a fatal untreatable disease involving degeneration of motor neurons. Μultiple causative genes encoding proteins with versatile functions have been identified indicating that diverse biological pathways lead to ALS. Chemical entities still represent a promising choice to delay ALS progression, attenuate symptoms and/or increase life expectancy, but also gene‐based and stem cell‐based therapies are in the process of development, and some are tested in clinical trials. Various compounds proved effective in transgenic models overexpressing distinct ALS causative genes unfortunately though, they showed no efficacy in clinical trials. Notably, while animal models provide a uniform genetic background for preclinical testing, ALS patients are not stratified, and the distinct genetic forms of ALS are treated as one group, which could explain the observed discrepancies between treating genetically homogeneous mice and quite heterogeneous patient cohorts. We suggest that chemical entity‐genotype correlation should be exploited to guide patient stratification for pharmacotherapy, that is administered drugs should be selected based on the ALS genetic background.

## 1 INTRODUCTION

Amyotrophic lateral sclerosis (ALS) (Table 1) is a rare but rapidly progressive neurodegenerative disease with an estimated prevalence of 2:100 000. It is characterised by loss of motor neurons in the brain and spinal cord, uniformly leading to death within 3–5 years from diagnosis, most frequently, due to respiratory paralysis.^1^ Two ALS types can be distinguished, that is familial ALS (FALS) that accounts for 5–10% of all ALS cases and sporadic ALS (SALS) that accounts for the rest. Ηallmark of ALS is the abnormal accumulation of intracellular protein aggregates that vary in composition and could contain TDP‐43, SOD1, FUS or various poly‐dipeptides encoded by pathogenic GGGGCC expansions in the *C9ORF72* gene. TDP‐43 aggregates in the cytoplasm of neuronal cells are a common finding in ALS (especially SALS) patients except for patients with pathogenic variants in *SOD1*.^2^ Intriguingly though, pathogenic variants in >30 different genes have been linked to ALS.^3^ In addition, disease modifier genes significantly increase the heterogeneity of ALS.^4^ Most commonly, mutations in the *C9ORF72*, *SOD1*, *TDP‐43* and *FUS* genes underlie respective FALS forms but mutations in these genes are also found in many SALS cases.^5^ The molecular complexity of SALS is further increased by various environmental factors^6^ highlighted by studies of amyotrophic lateral sclerosis‐parkinsonism‐dementia complex (ALS‐PDC) prevalent in the pacific island of Guam, outlined below.

**TABLE 1:** Abbreviations related to genetic forms of ALS and experimental ALS models

**Abbreviation**	**Full term**
**ALS**	Amyotrophic lateral sclerosis
**ALSFRS‐R**	Amyotrophic lateral sclerosis functional rating system‐revised
**ALS‐PDC**	Amyotrophic lateral sclerosis‐parkinsonism‐dementia complex
**C9ORF72**	Chromosome 9 open reading frame 72
**DPRs**	Dipeptide repeat proteins
**FALS**	Familial amyotrophic lateral sclerosis
**FTD**	Frontotemporal dementia
**FUS**	RNA‐binding protein fused in sarcoma
**RAN**	Repeat associated non‐ATG
**SALS**	Sporadic amyotrophic lateral sclerosis
**SMA**	Spinal muscular atrophy
**SOD1**	Superoxide dismutase 1
**TDP‐43**	Transactive response DNA binding protein 43
**TBK1**	Tank Binding Kinase 1
**Tg**	Transgenic
**Tg‐*C9ORF72***	Tg mouse carrying *C9ORF72* transgene with GGGGCC expansions
**Tg‐*PFN1^G118V^***	Tg mouse carrying transgene encoding for PFN^G118V^
**Tg‐*SOD1^G93A^***	Tg mouse carrying transgene encoding for SOD1^G93A^
**Tg‐*SOD1^G37R^***	Tg mouse or zebrafish carrying transgene encoding for SOD1^G37R^
**Tg‐*SOD1^G93A^Tbk1^+/–^***	Tg‐*SOD1^G93A^* mouse heterozygous for *Tbk1* knockout
**Tg‐*TDP‐43^A315T^***	Tg mouse carrying transgene encoding for TDP‐43^A315T^
**Tg‐*TDP‐43^G348C^***	Tg mouse carrying transgene encoding for TDP‐43^G348C^
**Tg‐*FUS^(1‐359)^***	Tg mouse carrying transgene encoding for truncated FUS 1–359
**Tg‐*FUS^S57Δ^***	Tg *C. elegans* carrying transgene encoding for FUS lacking S57
**Tg‐*FUS^R521H^***	Tg zebrafish carrying transgene encoding for FUS^R521H^
**Tg‐*EAAT2*/Tg‐*SOD1^G93A^***	Tg‐*SOD1^G93A^* mouse that also carries a transgene encoding for EAAT2
**Tg‐*TDP‐43ΔNLS***	Tg mouse carrying transgene encoding TPD‐43 that lacks the nuclear localisation signal
**Tg‐*TDP‐43ΔNLSMmp9^–/–^***	Tg‐TDP‐43ΔNLS mouse knockout for Mmp9

To address the vast heterogeneity of ALS, *omics* approaches were exploited for molecular taxonomy, especially, of SALS.^7^ For example, current analysis of transcriptomics data identified three distinct subtypes of ALS: one linked to retrotransposon activation, another in which oxidative stress is implicated, and a third characterised by activated glia.^8^ *Omics* can also reveal new disease pathways and candidate targets for pharmacological intervention.^9^

**FIGURE 1:**
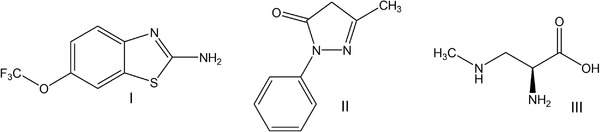
Chemical formulas of riluzole (I), edaravone (II) and β‐methylamino‐L‐alanine (L‐BMAA) (III)

Riluzole (**I**) and edaravone (**II**) (Figure 1) are the only drugs approved by the FDA for ALS; nevertheless, a very small improvement of disease or quality of life of ALS patients was observed by either of these drugs.^10^ Specifically, riluzole increased the lifespan by approximately 3 months, while edaravone could delay disease progression at the early stages.^10^ Stem cell therapies,^11^ gene therapies^12^ and vaccinations^13^ are currently under development, and some have entered clinical trials as for example the trials NCT00748501 and NCT01640067. Further, oligonucleotide‐based therapies for specific ALS subgroups have entered clinical trials^14^ that is, tofersen (BIIB067), an antisense drug targeting *SOD*1 (NCT02623699)^15^ and BIIB078 to be used in *C9ORF72*‐ALS patients (NCT03626012). Nevertheless, there is still a pressing need for chemical entities with pharmacological efficacy to attenuate disease symptoms and improve quality of life of ALS victims.

Chemogenomics involve the systematic analysis of the response(s) of a biological system to a chemical compound. Forward chemogenomics aims to unravel druggable targets by searching for molecules effecting a desirable phenotype, and reverse chemogenomics aims to identify molecules that bind to a given target.^16^ The term is used here to describe the use of a given chemical entity to treat a certain ALS genetic background.

### 1.1 The need for stratification of ALS patients

**FIGURE 2:**
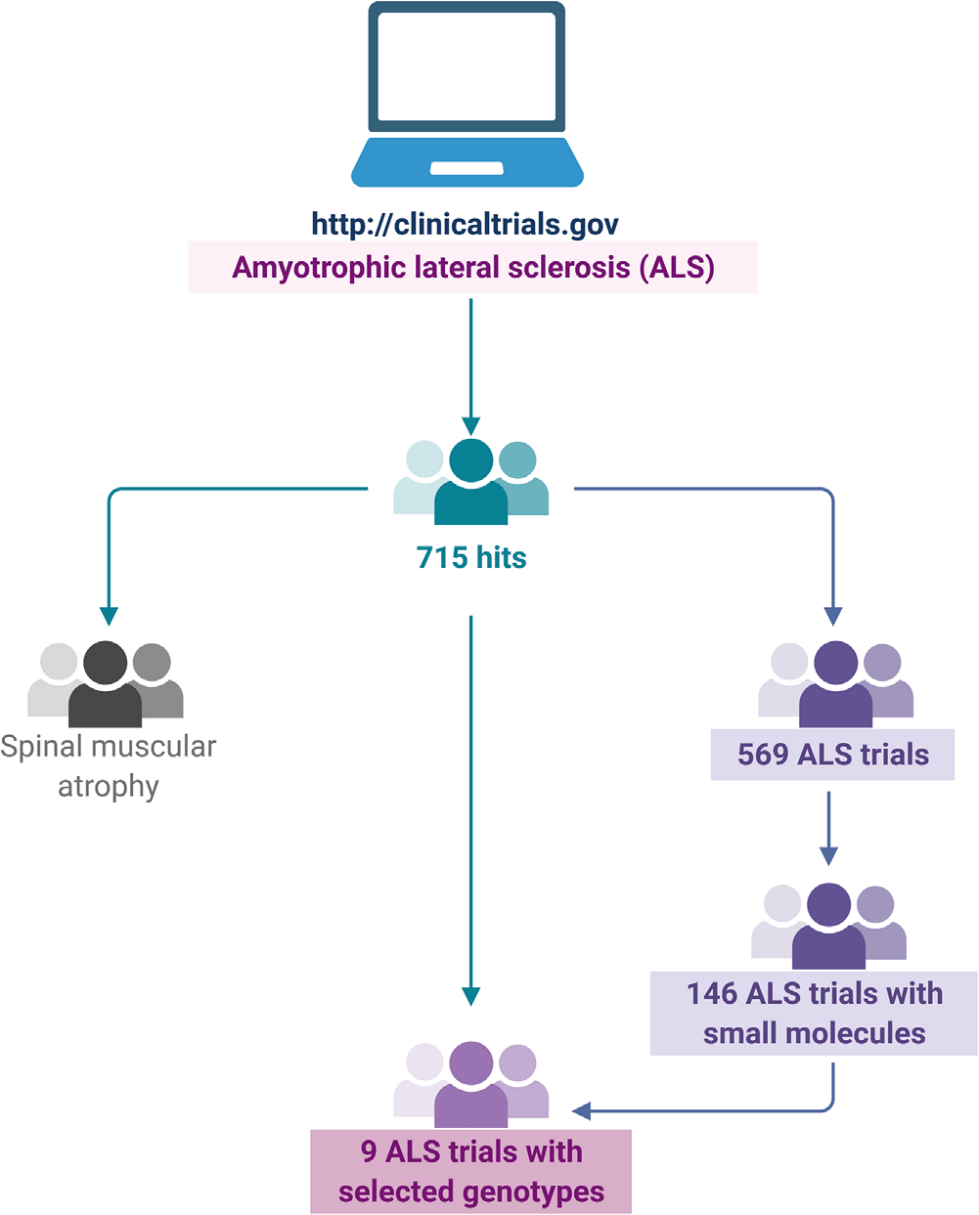
Flowchart of search in clinical trials. Interrogation of clinical trials (http://clinicaltrials.gov) against ‘amyotrophic lateral sclerosis’ retrieved 715 hits for ALS and the rest were for spinal muscular atrophy (SMA). Of ALS trials 146 involved the administration of 50 small molecules and only nine trials involved patients selected for the presence of a certain pathogenic variant. The image was created with Biorender (http://biorender.com)

There are eight distinct clinical features in ALS that include: classic (Charcot's phenotype), bulbar, flail arm, flail leg, pyramidal, respiratory and pure lower or upper motor neuron.^17^ It is now understood that the pathological underpinnings of ALS are heterogenous^18^ and, most likely, quite distinct biological pathways are involved, as also indicated by the functional versatility of the proteins encoded by pathogenetic variants of the identified familial ALS genes. The frequent failure of candidate compounds tested for ALS in clinical trials, which proved effective in animal models, could be explained by the fact that animal models represent a given ALS genotype, while clinical trials are conducted with non‐stratified, thus, genetically heterogeneous patient populations. Interrogation of the clinical trial database (http://clinicaltrials.gov) against ‘amyotrophic lateral sclerosis’ retrieved only nine recent trials, in which patients were selected based on their involved ALS pathogenic gene (Figure 2). In the era of precision medicine, treatment decisions are guided by genetic/molecular data according to which patients are stratified into subgroups. Two anecdotal examples of environmental exposure linked to ALS exemplify the benefit of ALS treatment and/or prevention from subgrouping of patients.

1. **ALS due to mercury intoxication**. Incidentally, an SALS patient was identified who suffered ALS likely linked to mercury intoxication and was treated with a combination of the chelator 2,3‐dimercaptopropanesulfate and α‐lipoic acid for mercury cleansing that was expected to ameliorate symptoms. This case may turn out to be the first reported ALS cure.^19^ This is reminiscent of the loss of metal homeostasis reported in Alzheimer disease, for which either Zn^2+^ supplementation or Fe^3+^/Al^3+^ chelation therapies have been suggested.^20^ Thus, screening ALS patients for metal intoxication might provide an alternative strategy to treat, using chelating drugs, a subgroup of ALS patients in which ALS could be related with metal intoxication. Notably, metallomic analysis revealed uranium in the CSF of 47% of ALS patients examined in a Scandinavian study,^21^ suggesting that chelation treatment could be beneficial for this subgroup.
2. **The case of Guam – How changing diet can prevent ALS**. In Guam, the incidence of ALS was unexpectedly high, that is approximately 200/100 000, making it a rather common neurodegenerative disease. Neurotoxin β‐methylamino‐L‐alanine (L‐BMAA) (**III**), a component of cycad seeds consumed in Guam was identified as the ALS causing agent. Cycad seeds are consumed and bioaccumulated by flying foxes (bats) in the island which are popular food for locals. As a result, they intake large quantities of L‐BMAA.^22^ Intravenous injection of L‐BMAA induces a disease in rats that mimics ALS/PDC and is accompanied by appearance of cytosolic TDP‐43 aggregates.^23^ The incidence of ALS‐PDC is constantly declining in Guam, following diet changes towards restricted bat consumption.^24^

## 2 THERAPEUTIC APPROACHES BASED ON THE GENETIC BACKGROUND OF ALS PATIENTS

### 2.1 Strategies to treat ALS caused by SOD1 pathogenetic variants

**FIGURE 3:**
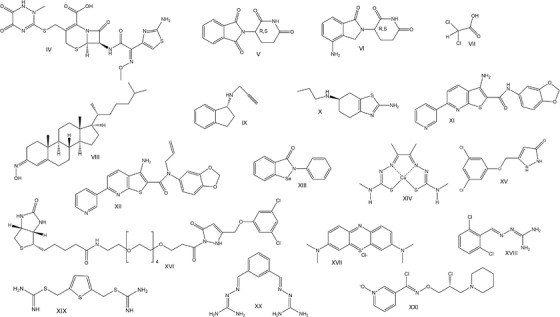
Chemical formulas of compounds used to treat *SOD1*‐ALS in disease models

Tg‐*SOD1^G93A^* mice represent the first animal model for ALS and remains the most widely used rodent model of human ALS.^25^ Chemicals that act on various biological pathways have been tested in these mice to investigate their putative effect on disease progression and overall survival (Figure 3). It is plausible though that the biological pathways in the ALS form recapitulated by Tg‐*SOD1^G93A^* mice are not implicated in all ALS subtypes.^26^ The chemical compounds used to treat *SOD1*‐ALS can be classified based on their mechanism of action, as outlined below.

#### 2.1.1 Mitigation of excitotoxicity

Excitotoxicity describes the neuronal damage caused by excessive stimulation due to glutamate accumulation in the synaptic cleft. ALS patients and mouse models show decreased levels of the excitatory amino acid transporter 2 (EAAT2) that is responsible for synaptic glutamate clearance.^27^, ^28^ Overexpression of *EAAT2* in Tg‐*SOD1^G93A^* mice significantly delays grip strength decline but does not alter the onset of disease symptoms or the lifespan of Tg‐*EAAT2*/Tg‐*SOD1^G93A^* mice compared to Tg‐*SOD1^G93A^*,^29^ indicating that EAAT2 overexpression accompanied by suppressed excitotoxicity is not the best option in this model. This is corroborated by clinical and preclinical data. For example, riluzole that mainly inhibits the release of glutamate^10^ could extend the lifespan of ALS patients by 2–3 months, only, while it had a modest effect in delaying disease progression.^30^ When administered at the onset of symptoms, it does not have any effect on lifespan or motor function of Tg‐*SOD1^G93A^*, Tg‐*TDP43^A315T^* and Tg‐*FUS^(1‐359)^* mouse models.^31^ The efficacy of riluzole in patients may also be compromised by its rapid metabolism by CYP1A2. Thus, prodrugs that withstand CYP1A2 metabolism and have increased in vivo stability were designed.^32^ Since serum levels of administered riluzole in patients are determined by the expression of CYP1A2,^32^ another way to increase the likelihood of response to riluzole could be to select ALS patients with low CYP1A2 levels.

*Ceftriaxone* (**IV**), a cephalosporine antibiotic, that increases the activity of the *EAAT2* gene promoter resulting in elevated EAAT2 expression that, in turn, reduces glutamate excitotoxicity,^33^ significantly improved the ALS phenotype of Tg‐*SOD1^G93A^* mice and extended their lifespan by 10 days.^34^ Consistently, earlier case studies had reported improvement of symptoms in some ALS patients upon administration of ceftriaxone.^35^ A later clinical trial (NCT00349622) showed no beneficial effect of ceftriaxone in non‐stratified ALS patients. Whether *SOD1*‐ALS patients were included in this trial is unknown as no genetic data are available for retrospective analysis, to validate whether ceftriaxone was beneficial in this subgroup.^33^ Nonetheless, in these trials there was no effort to ascertain target engagement (i.e. EAAT2 upregulation in people who received ceftriaxone) or neuronal hyper excitability (via TMS, for instance). Consequently, they should not be interpreted as indicators of the ineffectiveness of therapies targeting EAAT2.

#### 2.1.2 Targeting inflammation

TNFα is a major pro‐inflammatory cytokine with a wide variety of biological responses including the apoptosis of neuronal cells.^36^ Increased levels of TNFα and FasL have been found in biopsy sections of lumbar spinal cord from ALS patients (FALS‐*SOD1^I113T^* and SALS) and Tg‐*SOD1^G93A^* mice.^37^ Thus, thalidomide (**V**) and lenalidomide (**VI**), which inhibit TNFα production, were tested in Tg‐*SOD1^G93A^* mice. When administered pre‐symptomatically, both compounds improved motor performance, attenuated weight loss and extended the lifespan by approximately 3 weeks (16% and 18.5% increase in mean survival, respectively).^37^ When administered at the onset of symptoms, lenalidomide improved motor performance, attenuated weight loss and extended the lifespan of Tg‐*SOD1^G93A^* mice by approximately 19 days.^38^ A clinical trial with non‐stratified ALS patients found that thalidomide does not improve the ALSFRS‐R (ALS Functional Rating System‐Revised) score or the forced vital capacity (FVC).^39^ Thus, these compounds could be effective in *SOD1*‐ALS only, given their beneficial effect in Tg‐*SOD1^G93A^* mice. To this end, it is interesting to note that deletion of *Tnfα* in Tg‐*SOD1^G93A^* and Tg‐*SOD1^G37R^* mice did not increase the lifespan and did not inhibit the extent of neuronal loss^40^ indicating that, besides than TNFα‐inhibition, thalidomide and lenalidomide, may have other (off‐target) functions in vivo probably linked to the extended lifespan of Tg‐*SOD1^G93A^* mice. Mapping the off‐target effects in mice may reveal novel targets for pharmacological intervention. This endeavour could be accelerated or advanced by use of drug‐based activity‐based probes (ABPs), as will be described below. Nevertheless, well‐known side‐effects of thalidomide and lenolidomide, as for example, the sensory and motor axonal neuropathy^41^, ^42^ could complicate their use for ALS, a motor neuron disease, and could account for their failure in clinical trials.

#### 2.1.3 Mitochondria targeting

Mitochondria dysfunction is a characteristic feature of ALS.^6^ Tg‐*SOD1^G93A^* mice show decreased respiratory capacity in astrocytes. Dichloroacetate (DCA) (**VII**) is a pyruvate dehydrogenase kinase inhibitor that stimulates mitochondrial metabolism.^43^ When administered in drinking water of Tg‐*SOD1^G93A^* mice, it extended survival by 2 weeks in males and by 10 days in females, while it improved grip strength.^44^

*Olesoxime* (**VIII**) is a mitochondrial pore modulator that reduces neuronal cell death in Tg‐*SOD1^G93A^* mice^45^; nevertheless, a Phase II/III clinical trial did not validate these positive effects in non‐stratified ALS patients.^46^

*Coenzyme Q10*, a compound that has been shown to improve mitochondrial function in humans when administered orally,^47^ slightly increases the lifespan of Tg‐*SOD1^G93A^* mice,^48^ but a clinical trial with non‐stratified ALS patients (NCT00243932) could not validate significant improvement of ALS phenotype for a high dose of coenzyme Q10.^49^ Other dietary changes to alleviate ALS symptoms have also been tested. The Deana Protocol Supplement involves arginine α‐ketoglutarate, γ‐aminobutyric acid (GABA), coenzyme Q10 and medium chain triglycerides. When administered to 10‐week‐old Tg‐*SOD1^G93A^* mice, this supplement improved survival and motor functions.^50^ Similarly, caprylic triglyceride, which was used in a recent clinical trial (NCT02716662), enhanced motor performance in different tests, increased mitochondrial respiration compared to controls, but could not extend survival. Also, vitamin E delayed the onset of disease symptoms in Tg‐*SOD1^G93A^* mice but did not increase the lifespan.^51^ In a large clinical trial, high doses (5 g per day) of vitamin E did not have significant beneficial effects on survival or alleviation of symptoms.^52^ The SS31 antioxidant cell‐permeable peptide (D‐Arg‐dimethyltyrosine‐Lys‐Phe‐NH_2_) that targets the inner mitochondrial membrane, improved survival and motor performance in *Tg‐SOD1^G93A^* mice and reduced neuronal cell apoptosis induced by hydrogen peroxide^53^ but it has not yet been validated in clinical trials. Since the aforementioned supplements proved effective in Tg‐*SOD1^G93A^*, their therapeutic effect may be limited to *SOD1*‐ALS patients.

*Rasagiline* (**IX**), a MAO‐B inhibitor that has antioxidant and anti‐apoptotic functions,^54^ extends the lifespan and improves the running wheel performance of Tg‐*SOD1^G93A^* mice. Co‐administered with riluzole, *rasagiline* showed additive effects in Tg‐*SOD1^G93A^* mice.^55^ Currently, rasagiline is being tested in ALS patients of various disease genotypes (NCT01879241, NCT01786603), with positive results in reducing oxidative stress in mitochondria, and increasing the mitochondrial membrane potential.^56^ *Dexpramipexole* (**X**), a low‐affinity binding compound for dopamine receptors, had protective effects in vitro manifested by improved mitochondrial function, prevented apoptosis and reduced ROS,^57^ but it failed to exhibit any positive results in Tg‐*SOD1^G93A^* mice and, in a Phase III clinical trial in ALS patients.^58^

Due to their anti‐excitotoxicity, antioxidant and anti‐inflammatory actions, cannabinoids have been extensively tested in Tg‐*SOD1^G93A^* mice, and a recent meta‐analysis showed that they increase mice survival by 3.84 days.^59^ In one study, the cannabinoid CB2 selective agonist HU‐308 was administered in Tg‐*TDP‐43^A315T^* mice and was found to improve rotarod performance but had no effect on survival.^60^ A clinical trial with ALS patients treated with cannabis oil is ongoing.^61^ On the other hand, Klotho was demonstrated to delay the onset of disease symptoms and to increase the lifespan of both male and female Tg‐*SOD1^G93A^* mice, in which the beneficial effects were more pronounced.^62^ Finally, GNX‐4728, a cinnamic anilide derivative that acts as inhibitor of the mitochondrial permeability transition pore has been tested in Tg‐*SOD1^G37R^* male mice and was found to increase their lifespan from 366 to 686 days (mean values) and to delay the onset of symptoms.^63^

#### 2.1.4 Targeting SOD1 aggregation

Formation of SOD1 aggregates can be suppressed either by direct inhibition of SOD1 aggregation or by suppression of *SOD1* expression,^32^ as for example with pyrimethamine. In *SOD1*‐ALS patients, pyrimethamine lowers the levels of SOD1 in CSF.^64^ A clinical trial was designed that solely included *SOD1*‐ALS patients, in which the levels of SOD1 in CSF were monitored (NCT01083667). The effect on disease progression has not been investigated.

Another strategy to diminish the formation of aggregates involves the stabilisation of SOD1 dimers through chemical crosslinking between adjacent Cys^111^ of two SOD1 molecules with maleimide derivatives and thiol‐disulfide exchange approaches, for example, with 1,4‐bismaleimidobutane.^65^ A recent strategy targets the interaction between SOD1 and derlin‐1, which plays a role in the endoplasmic reticulum (ER) machinery.^66^ Disrupting this interaction alleviated ALS symptoms.^67^ High‐throughput screening (HTS) identified compound **XI** that prevents the interaction of mutant SOD1 with derlin‐1. A series of analogues were synthesised and **XII** with better physicochemical properties was tested as a candidate drug. **XII** alleviated pathology of Tg‐*SOD1^G93A^* mice and of motor neurons derived from iPSCs of patients with *SOD1*‐ALS.^67^

Screening of 640 FDA‐approved drugs found that statins (simvastatin, lovastatin, mevastatin) and vitamin D3 derivatives (alfacalcidol, calcidiol, calcitriol) inhibited aggregation of apo‐SOD1^G37R^.^68^ Unexpectedly, statins accelerate disease progression, decrease the lifespan of *Tg‐SOD1^G93A^* mice^69^ and worsen the phenotype of ALS patients manifested by increased rates of ALSFRS‐R decline and higher frequency of muscle cramps, although the latter may associate with statin‐induced myopathy.^70^ These observations uncover the limitations of HTS against pure components, like the SOD1 aggregates, in the effort to identify new drugs, but vitamin D3 supplementation in Tg‐*SOD1^G93A^* mice improves motor function.^71^ Nevertheless, although earlier studies showed that patient intake of vitamin D3 slowed the rate of ALSFRS‐R decline,^72^ later studies did not replicate these findings.^73^, ^74^ These contradictory clinical evidences may be due to the quite varying genetic background of recruited patients.

*Ebselen* (**XIII**) is an organoselenium cysteine reactive compound that promotes the formation of the intramolecular disulfide bond of SOD1 and its correct folding.^75^ Thus, it enhances the formation of the functional SOD1 dimer instead of the toxic aggregates. In addition, ebselen has antioxidant activity.^75^ Although it only marginally extended the lifespan of Tg‐*SOD1^G93A^* mice, it significantly delayed the onset of disease symptoms.^76^ Thus, it may alleviate symptoms in *SOD1*‐ALS patients.

In analogous manner, treatment with copper diacetyl‐di(N4‐methyl)thiosemicarbazone (Cu(II)ASTM) (**XIV**) improves symptoms and extends survival in Tg‐*SOD1^G93A^* mice^77^ and Tg‐*SOD1^G37R^* mice.^78^ A mechanism that could account for this function involves copper transfer to SOD1. Thus, loading SOD1 with metal ions may provide a new type of *SOD1*‐ALS‐specific therapy. A Phase I clinical trial to assess the pharmacokinetics of Cu(II)ASTM in ALS patients has been completed (NCT02870634) and another study (NCT04082832) is ongoing to assess its efficacy without; however, selecting for *SOD1*‐ALS patients in which drug efficacy has been proven in the respective mouse model.

Finally, *pyrazolone* derivatives have been identified as SOD1 aggregation inhibitors.^79^, ^80^ Pyrazolone **XV** increased the lifespan of Tg‐*SOD1^G93A^* by 13%.^81^ In this case, an activity‐based probe (ABP) was designed to map its complete interactome (i.e. biological targets and off‐targets). For this, a biotin moiety with a spacer was introduced onto the one side of the molecule and the new derivative (**XVI**) was immobilised on streptavidin beads. The beads were incubated with cellular lysates and the precipitants were analysed by mass spectrometry to identify the biological targets.^82^ This assay revealed an unexpected function of pyrazolones, which is proteosomal activation without heat shock response.

#### 2.1.5 Targeting proteolysis

Biochemical pathways involving proteases may provide novel targets for ALS treatment. In this respect, the widely studied metalloprotease MMP9 mediates the degeneration of fast motor neurons in Tg‐*SOD1^G93A^* mice through enhancement of ER stress^83^ and/or regulation of Tnfα and FasL expression.^84^ Deletion of one *Mmp9* allele in Tg‐*SOD1^G93A^* increased the lifespan by 14% and deletion of both *Mmp9* alleles (Tg‐*SOD1^G93A^Mmp9^–/–^*) by 25%, which is one of the longest prolongations of lifespan ever reported for an intervention in any of ALS mouse models. Intracerebroventricular (icv) administration of an MMP9 inhibitor in Tg‐*SOD1^G93A^* mice delayed denervation and reduced ER stress.^83^ Knockdown or knockout of MMP9 attenuates the neuromuscular defects in rNLS8 (Tg‐*TDP‐43ΔNLS*) mice. Intriguingly though, Tg‐*TDP‐43ΔNLSMmp9^–/–^* mice have significantly shortened survival and run slower than the rNLS8, despite their attenuated neuromuscular defects.^85^ Thus, targeting MMP9 for inhibition may represent a beneficial therapeutic strategy only for *SOD1*‐ALS patients.

#### 2.1.6 Maintenance of proteostasis

The dynamic regulation of a balanced, functional proteome (proteostasis) orchestrates multiple cellular systems and functions like the ubiquitin proteasome system, autophagy, ER stress/unfolded protein response, stress granules and heat shock proteins, to provide ‘quality control’ for proteome maintenance. The proteostasis drug *methylene blue* (**XVII**), that induces autophagy,^86^ could rescue motor defects in Tg‐*TDP‐43^A315T^* and Tg‐*FUS^S57Δ^ C. elegans* models and in Tg‐*TDP‐43^G348C^* and Tg‐*FUS^R521H^ D. rerio* ^87^ but not in *Tg‐TDP‐43^G348C^* ^88^ and Tg‐*SOD1^G93A^* mice.^89^ These results indicate that selection of the appropriate animal model is essential for extrapolation of animal data to putative effective therapies in the clinic. The scheme of administration, the time for initiation of treatment should also be optimised, as indicated by the discrepancies observed upon administration of methylene blue in *C. elegans* and *D. rerio* in which it was administered at hatching and proved effective, but not in Tg‐*SOD1^G93A^* mice, in which administration was initiated at 45 days or at 90 days and after 6 months from birth in Tg‐*TDP‐43^G348C^*.^87^, ^88^, ^89^

*Sephin 1* (**XVIII**), a closely related guanabenz analogue, is a selective inhibitor of the stress‐induced phosphatase PPP1R15A that prolongs eIF2 phosphorylation upon stress and protects cells from ER stress.^90^ In Tg‐*SOD1^G93A^* mice, sephin 1 prevents weight loss and improves motor function.^90^ The original compound guanabenz also inhibits PPP1R15A. Although it showed efficacy in Tg‐*SOD1^G93A^* mouse models,^91^, ^92^ it has severe side‐effects, since it also binds to the α2‐adrenergic receptor.^93^

Bis‐guanyhydrazones that act as molecular chaperones, such as the R55 (**XIX**), and affect proteostasis, have been developed for the potential treatment of ALS.^94^ Further, molecular optimisation of R55 led to the compound (**XX**) that was shown to slow down the progression of motor deficits, and to reduce the degeneration of nerve fibres and SOD1^G93A^ aggregation in Tg‐*SOD1^G93A^* mice.^95^

Another approach to target *SOD1*‐ALS involves the activation of heat shock family chaperones that protect cells from various stresses. Arimoclomol (**XXI**), a heat shock protein inducer, delays the onset of symptoms, extends the lifespan, promotes muscle function and prevents the aggregation of SOD1 in *Tg‐SOD1* ^G93A^ mice.^96^, ^97^ In the clinical trial NCT00706147, rapidly progressive *SOD1*‐mutant ALS patients were treated with up to 200 mg arimoclomol and this Phase II trial showed that arimoclomol is safe and well‐tolerated.^98^ A Phase III clinical trial (NCT03836716) with 231 recruited patients aims to define the long‐term safety and efficacy of arimoclomol in *SOD1*‐ALS patients.

### 2.2 Targeting G4C2 pathogenetic expansions in *C9ORF72*

The GGGGCC large repeat expansions (from 250 up to > 3000 repeats) present in the first intron of the *C9ORF72* gene and coding for poly‐dipeptides [poly(GA), poly(GP), poly(GR), poly(PR) and poly(PA)] have been identified in patients suffering from ALS with or without frontotemporal dementia (FTD).^99^, ^100^ These hexanucleotide expansions are transcribed bidirectionally and form either RNA foci in the nucleus, that binds and sequesters RNA‐binding proteins, or are translated into dipeptide repeat proteins (DPRs) by an abnormal translation process called repeat‐associated non‐ATG (RAN) translation. This results in gain‐of‐function that leads to neuronal cell toxicity.^101^, ^102^, ^103^, ^104^ Loss‐of‐function due to *C9ORF72* repeat expansion mutations in ALS was revealed in *C9orf72* ^–/–^ mice and included dysregulation of autophagy and membrane trafficking and development of inflammatory reactions.^105^

These GGGGCC repeats exhibit somatic instability; thus, it is possible that repeat expansion may not be present in DNA from blood samples although present in CNS. Therefore, a blood DNA test negative for GGGGCC expansion does not necessarily indicate that the patient is not a *C9ORF72^ExpGGGGCC^* carrier,^106^, ^107^ and this should be taken into consideration when classifying ALS patients. Evidently, a combination of genetic analysis and determination of poly(GP) in CSF will be required for a definite clinical diagnosis,^106^, ^107^ with apparent clinical implications. Approaches to alleviate symptoms or decelerate the course of the disease include targeting the RNA transcribed from GGGGCC‐repeats or targeting the DPRs. These RNAs adopt two folded states that are in equilibrium, a hairpin structure and the G‐quadruplex structure.^108^

#### 2.2.1 Targeting RNA G‐quadruplexes/hairpins

**FIGURE 4:**
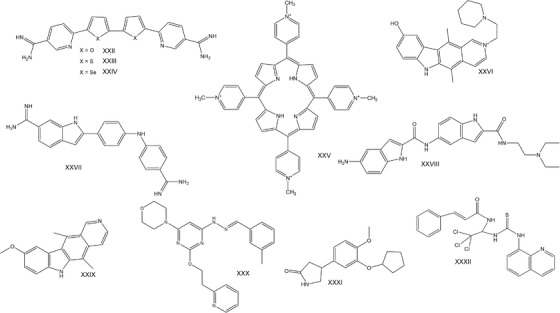
Chemical formulas of compounds used to treat *C9ORF72*‐ALS in disease models

HTS identified the structurally similar chemicals DB1246 (**XXII**), DB1247 (**XXIII**) and DB1273 (**XXIV**) (Figure 4) that exhibit high‐affinity binding on GGGGCC RNA G‐quadruplexes. These chemicals significantly reduced RNA foci in vitro in human iPSC‐motor and iPSC‐cortical neuron cell lines and decreased DPRs. Decreased levels of DPRs and improved survival of larvae reaching the pupal stage of development was observed in vivo, in GGGGCC repeat‐expressing *Drosophilas* treated with DB1273.^109^

The cationic (5,10,15,20‐tetra(N‐methyl‐4‐pyridyl) porphyrin (TMPyP4) **XXV** binds to GGGGCC RNA G‐quadruplexes in a concentration‐dependent manner and causes a conformational change in their secondary structures conferring thermal instability of GGGGCC RNA G‐quadruplexes. Thus, TMPyP4 blocks the interaction of GGGGCC RNA G‐quadruplexes with RNA‐binding proteins, such as ASF/SF2 and hnRNPA1.^110^ Another group developed three compounds (**XXVI**, **XXVII**, **XXVIII**) targeting the hexanucleotide repeat region of RNAs and tested them for binding to hairpin RNA and reducing RAN translation in a cell‐free model. **XXVI** and **XXVII** reduced RNA foci and significantly decreased RAN translation in GGGGCC repeat‐expressing neurons.^108^ Improving the selectivity of **XXVI** led to compound **XXIX** that binds selectively in the internal loops of the hairpin form of RNA. **XXIX** blocked polysome assembly and reduced RNA foci and RAN translation in vitro.^111^ The in vivo action of these compounds in *C9ORF72*‐ALS remains to be validated.

#### 2.2.2 Targeting DPRs

RAN translation of *C9ORF72* RNA GGGGCC‐repeats in all six‐reading frames produces five repeated polypeptides [poly(GA), poly(GP), poly(GR), poly(PR) and poly(PA)] from which mainly the poly(GR), poly(PR) and poly(GA) are toxic to cells through induction of nucleolar stress and defects in mRNA splicing.^103^, ^104^, ^112^

Inhibition of PIKFYVE kinase that converts phosphatidylinositol‐3‐phosphate (PI3P) to phosphatidylinositol‐3,5‐biphosphate (PI(3,5)P2) leads to increased PI3P levels that regulate autophagosome formation and engulfment of proteins for degradation. Therefore, it may be important for the destruction of DPRs. Indeed, the PIKFYVE inhibitor apilimod (**XXX**) that was originally identified as a therapeutic compound in iPSC motor neurons derived from *C9ORF72*‐ALS patients^113^ reduces DPRs in C9‐BAC mice that harbour the human *C9ORF72* with 100–1000 GGGGCC repeats.^114^

Another way to reduce protein aggregates, including DPRs, is through proteosome activation. Rolipram (**XXXI**) is an antidepressant drug that acts via inhibition of phosphodiesterase 4 (PDE4) and promotes proteasome function.^115^ Rolipram decreased poly(GA) in primary hippocampal neurons in vitro.^116^ DPRs induce integrated stress response (ISR) through ER implicating *TMX2*.^117^ ISR involves hyperphosphorylation of elf2a and increased RAN of *C9ORF72* RNA GGGGCC‐repeats.^118^ **XXXII**, a selective inhibitor of eIF2α dephosphorylation and tauroursodeoxycholate (TUDCA), a chemical chaperone, showed protection against poly(GA)‐induced stress and cell death in vitro.^119^ In conclusion, many compounds that can target the DPRs are available, which could be further validated in clinical trials. DPR aggregation could be assessed in Tg‐*C9ORF72* mice, nevertheless, these mice display no behavioural or survival differences compared to wt mice.^120^

### 2.3 Targeting *TDP‐43* pathogenic variants

Transactive response DNA binding protein 43 (TDP‐43) is primarily a nuclear protein that binds to UG/TG repeats in the introns of pre‐mRNA^121^ and regulates transcription or RNA processing.^122^ Under stress conditions, TDP‐43 is thought to localised in stress granules,^123^ although other studies have shown that TDP‐43 forms cytoplasmic aggregates that are distinct from stress granules in that they do not contain RNA.^124^, ^125^ These cytoplasmic aggregates consist of full‐length TDP‐43 or of its C‐terminal proteolytic fragments of 35 and 25 kDa.^126^ Further, cytoplasmic TDP‐43 is mainly ubiquitinated and phosphorylated in ALS patients.^127^ Recently, it was shown that loss of TDP‐43 results in unmasking of a cryptic exon and in introduction of a premature polyA tail in the *STMN2* mRNA that yields truncated stathmin‐2. This truncated transcript is absent in *SOD1*‐ALS.^128^ The involvement of *STMN2* in ALS is also corroborated by the recent finding that a long CA repeat polymorphism associates with increased risk and early onset of ALS in North American population.^129^ TDP‐43 targeting for therapeutic intervention could be achieved by the inhibition of phosphorylation or proteolytic cleavage or aggregation of TDP‐43, alternatively, by induction of autophagy and proteasome activation to clear the misfolded and/or aggregated TDP‐43.^130^

#### 2.3.1 Targeting autophagy or proteasome

TDP‐43 regulates the production of ATG7, an autophagy mediator, by stabilising the *ATG7* mRNA. Thus, depletion of TDP‐43 causes loss of ATG7 and impaired autophagy.^131^ Furthermore, loss of TDP‐43 increases TFEB nuclear translocation and enhances autophagosomal and lysosomal biogenesis but it impairs fusion of autophagosomes with lysosomes.^132^ Consequently, TDP‐43 aggregation observed in ALS could lead to dysregulation of autophagy. In addition, the 25 kDa TDP‐43 fragment causes severe cognitive and behavioural deficits in mice, and suppression of autophagy and proteasome activation.^133^

Inhibitors of mTOR act as activators of autophagy through induction of autophagosome formation.^134^ Administration of the autophagy activators rapamycin, spermidine, carbamazepine and tamoxifen in a mouse model of ALS/FTD with TDP‐43 aggregates decreased the loss of motor neurons and TDP‐43 aggregates.^135^ Rapamycin also increased the lifespan of a *Drosophila* ALS/FTD model.^136^ In contrast, in Tg‐*SOD1^G93A^* mice, rapamycin decreased survival and induced mitochondrial dysfunction and cell death in cultured motor neurons isolated from these animals.^137^ The above data further support the hypothesis that different therapies should be used for different ALS subtypes.

A HTS study found fluphenazine (FPZ), methotrimeprazine (MTM) and 10‐(4′‐(N‐diethyl amino)butyl)‐2‐chlorophenoxazine (NCP) as the most potent autophagy activators. When neuronal and astrocyte ALS cell models were treated with these chemicals, the levels of TDP‐43 aggregates were reduced, the cell survival was improved and cytoplasmic mislocalisation of TDP‐43 was prevented.^138^

#### 2.3.2 Phosphorylation of TDP‐43

**FIGURE 5:**
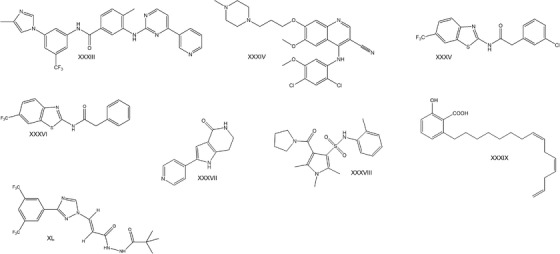
Chemical formulas of compounds used to treat *TDP‐43*‐ALS in disease models

Casein kinase 1 (CK‐1), cell division cycle 7 (CDC7), tau and tubulin kinase 1 and 2 (TTBK1 and TTBK2, respectively) and mitogen‐activated protein kinases (MAPK/ERK) phosphorylate TDP‐43 that in turn promotes the aggregation in the cytoplasm.^139^ Nilotinib (**XXXIII**) and bosutinib (**XXXIV**) (Figure 5), two tyrosine kinase inhibitors (TKI), altered glutamate synaptic signalling and showed neuroprotective effects in Tg‐*TDP‐43^wt^* mice.^140^ Also, nilotinib reversed mitochondrial dysfunction caused by TDP‐43 aggregates.^140^ In addition, bosutinib increased survival of iPSC‐derived motor neurons from patients with SALS or FALS caused by mutations in TDP‐43 or GGGGCC repeat expansions in *C9ORF72* while it delayed disease onset in Tg‐*SOD1^G93A^* for 11 days and moderately increased survival by 8 days.^141^ Since Tg‐*SOD1^G93A^* mice do not show TDP‐43 pathology, the latter finding also indicates that these compounds could have additional effects besides inhibiting TDP‐43 phosphorylation.^141^ Thus, TKIs could represent a broad therapeutic strategy for ALS since they showed clinical efficacy in genetically diverse ALS models.

A series of *N*‐(benzothiazolyl)‐2‐phenyl‐acetamide compounds were developed and optimised for CK‐1δ inhibition. The compound **XXXV** exhibited the lowest IC_50_ of 23 nM, while the **XXXVI** had an IC_50_ of 47 nM and both could penetrate the blood brain barrier (BBB). **XXXV** decreased phosphorylation of TDP‐43 and increased the lifespan of the Tg‐*TDP‐43 Drosophila*.^142^ Both **XXXV** and **XXXVI** prevented TDP‐43 phosphorylation and mislocalisation shuttling in *PGRN*‐(progranulin) deficient lymphoblasts^143^ and in lymphoblasts from SALS patients (negative for SOD1 pathogenetic variants and one positive for expansions in *C9ORF72*).^144^ The CDC7 selective inhibitor PHA767491 (**XXXVII**) abolished phosphorylated forms of TDP‐43 in vitro and in vivo in *C. elegans*,^145^ indicating a possible target against pathological phosphorylation of TDP‐43.

#### 2.3.3 Targeting cytoplasmic aggregates

Screening of 35 kinase inhibitors against paraquat‐treated SH‐SY5Y cells identified inhibitors of cyclin‐dependent kinases (CDKs) and glycogen synthase kinase 3 (GSK3) to block cytoplasmic TDP‐43 accumulation, thus alleviating intracellular cell stress.^146^ Another chemical discovered by HTS, the LDN‐0130436 (**XXXVIII**), improved the motor behavioural deficits of Tg‐*TDP‐43^wt^* and Tg‐*TDP‐43^A315T^ C. elegans*.^147^

#### 2.3.4 Other compounds

Various attempts aimed to identify compounds that may display therapeutic effect in TDP‐43‐ALS. In a screening of 1200 FDA‐approved drugs, the PPARγ agonist pioglitazone was identified to improve the locomotor function of Tg‐*TDP‐43^wt^* or Tg‐*TDP‐43^G298S^ Drosophilas*, yet it did not improve the survival of flies.^148^ Unfortunately, when pioglitazone was used in an ALS clinical trial in combination with riluzole (NCT00690118), it did not increase patient survival neither it improved any of the clinical symptoms.^149^ Failure of pioglitazone may be related to the fact that it either acts on certain ALS subtypes or the above‐mentioned *Drosophila* models do not recapitulate the corresponding human ALS subtypes. This resembles the already mentioned case of methylene blue that displays pharmacological activity in *C. elegans* models of ALS but not in mouse models.

Anacardic acid (**XXXIX**) acts as a histone acetyltransferase inhibitor and decreases *TDP‐43* mRNA and protein levels in human iPSCs derived from ALS patients carrying *TDP‐43* pathogenetic variants.^150^ A different strategy for treating TDP‐43 associated ALS involves targeting nuclear exportins that control TDP‐43 mislocalisation. TDP‐43 has a putative nuclear export signal (NES) recognised by XPO1. Although some studies have refuted it,^151^, ^152^, ^153^ the selective inhibitor of nuclear export (SINE) that targets XPO1, KPT‐350 (**XL**) partially rescues the motor deficits in a rat model of ALS/FTD generated by adenoviral delivery of *TDP‐43*.^151^

Recently, overexpression of the chaperone Sigma‐1 receptor in *Drosophila* models of C9‐ALS significantly reduced the neurodegenerative symptoms manifested by necrotic spots in the eyes by more than 12‐fold compared to controls.^154^ Deletion of *Sigma‐1* receptor encoding gene in Tg‐*SOD1^G93A^* mice exacerbated disease and shortened their life expectancy by approximately 30%.^155^ In the future, activators of Sigma‐1 receptor could be exploited for treatment of *SOD1*‐ALS and C9‐ALS.

### 2.4 FUS mutations

**FIGURE 6:**
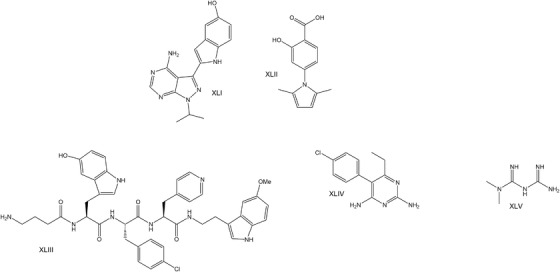
Chemical formulas of compounds target *FU*S‐ALS or gene modifiers

Patients with *FUS* mutations are characterised by absence of TDP‐43 aggregates.^156^ The treatment of the subpopulation of patients with *FUS*‐ALS is currently based on autophagy induction and alleviation of oxidative stress. Torkinib (**XLI**) (Figure 6) inhibition of mTOR induces autophagy more potently than rapamycin and reduces TDP‐43^P525L^ aggregates in engineered iPSC‐derived spinal neurons induced by arsenite. Torkinib improved dose‐dependently the motor neuron dysfunction in three FUS *Drosophila* models (FUS^wt^, FUS^R521C^, FUS^P525L^) measured with the climbing assay.^157^ Mutant FUS‐aggregates are localised in stress granules and stimulating autophagy with rapamycin decreases FUS in these granules.^158^ Furthermore, mutant FUS is implicated in the first stages of autophagosome formation and Rab1 overexpression restores autophagy function.^159^ In a controlled ongoing clinical trial (NCT03707795), betamethasone is used in ALS patients with *FUS* mutations to alleviate oxidative stress and improve symptoms.

### 2.5 The role of *TBK1* in ALS

Pathogenic variants in Tank Binding Kinase 1 (*TBK1*) gene implicated in autophagy regulation have been linked with FALS.^160^ Interestingly, co‐occurrence of TBK1 mutations with variants in other ALS genes has been detected in single ALS patients.^161^ Deletion of *Tbk1* in mice leads to embryonic lethality but *Tbk1^+/–^* mice are viable.^162^ To investigate the effect of *Tbk1* loss in the presence of other ALS‐related genes, mouse models of ALS have been generated on the *Tbk1^+/–^* background, like Tg‐*TDP‐43^G298S^Tbk1^+/–^*, that developed more severe pathology although life expectancy was not significantly altered.^163^ Despite the fact that symptoms developed earlier in Tg‐*SOD1^G93A^Tbk1^+/–^* than in Tg‐S*OD1^G93A^*, the Tg‐*SOD1^G93A^Tbk1^+/–^* had longer life expectancy, which may indicate differential effects of Tbk1 in early and late stages of ALS.^164^ TBK1 is an endogenous inhibitor of receptor‐interacting Ser/Thr protein kinase 1 (RIPK1) and an age‐related activation of RIPK1 is observed in *TBK1*‐ALS patients that leads neuroinflammation and neurodegeneration.^165^ RIPK1 inhibitors, specifically the DNL474 (the chemical formula has not been disclosed), are in clinical trials for ALS (NCT03757351).^166^ In addition, RIPK1 may be implicated in various ALS subtypes that are due to pathogenic *SOD1*, and*OPTN* (optineurin) variants.^167^

## 3 PHARMACOLOGICAL MODULATION OF GENE MODIFIERS

Modifier gene alleles either exacerbate or attenuate the clinical presentation of ALS (Table 2). The identification and characterisation of modifier genes is an ongoing endeavour that could provide new therapeutic options for certain ALS subgroups.^168^ Ephrin A4 (EPHA4) is a well‐established ALS gene modifier.^169^ Loss‐of‐function mutations in *EPHA4* associate with longer survival of ALS patients in different ALS animal models (Table 2). Thus, pharmacological inhibition of EPHA4 could provide a new way to treat ALS. In this direction, the 4‐(2,5‐dimethyl‐1H‐pyrrol‐1‐yl)‐2‐hydroxy benzoic acid (**XLII**), a pharmacological inhibitor of EphA4,^170^ rescues mutant SOD1‐induced axonopathy in zebrafish.^169^ The compound 123C4 (**XLIII**) is an EphA4 receptor binding agent that prolongs survival of Tg‐*SOD1^G93A^* mice by 8.5 days.^171^ EPHA4 can be considered a ‘universal’ ALS gene modifier since it acts as modulator for both SOD1 and TDP‐43 associated ALS. However, administration of antisense oligonucleotides targeting *EphA4* did not affect motor function or survival of Tg‐*SOD1^G93A^* or Tg‐*PFN1^G118V^* mice, although it significantly delayed (from 154 to 199 days) the onset of symptoms in Tg‐*PFN1^G118V^*.^172^ It should be mentioned that Tg‐*PFN1^G118V^* mice carry a transgene encoding for the G118V variant of profilin 1 gene (*PFN1*) that has been associated with rare cases of FALS.^173^ The success of chemical targeting of EphA4 to prolong survival in animal models over oligonucleotide targeting could be related to the fact that it targets Eph4 both to the CNS and to the periphery, while oligonucleotides administered icv target only the CNS.

**TABLE 2:** Gene modifiers for ALS

**Gene**	**Effect on ALS**	**Method/model**	**Subtype of ALS**	**Reference**
*EPHA4*	Loss of function increases survival in mice, zebrafish, patients	*TDP‐43^A315T^* zebrafish	TDP‐43, SOD1	^169^
	Pharmacological inhibition increases survival	*SOD1^A4V^, SOD1^G93A^, SOD1^G37R^* zebrafish		
		Tg‐*SOD1^G93A^EphA^+/–^* mice		
		Pharmacological targeting in Tg‐*SOD1^G93A–^* rat		
*EPHA4*	Extends survival	Pharmacological targeting in Tg‐*SOD1^G93A^* mice	SOD1	^171^
*EPHA4*	Delays onset	Tg‐*PFN1^G118V^* mice	PFN	^173^
*CX3CR1*	249I/I and 249V/I genotypes associate with shorter survival	Patients		^175^
*CX3CR1*	Knockout reduces survival	Tg*SOD1^G93A^ Cx3cr1^–/–^* mice	SOD1	^176^
*IL6R*	C variant Asp358Ala Increased rate of progression	Patients		^177^
*KCNJ11*	Rs5219 increased survival in bulbar ALS, patients with T/T survived longer	Patients		^202^
*ABCC8*	Rs4148646 increased survival in bulbar ALS patients with G/G survived longer, Rs4148642	Patients		^202^
	In spinal ALS patients with C/C have increased progression rate			
*UNC13A*	Rs12608932 associates with shorter survival	Patients		^203^

Lithium carbonate (Li_2_CO_3_) increased the 12‐month survival probability of ALS patients bearing the *UCN13A* C/C polymorphism. Thus, lithium carbonate may be used only for this patient subgroup.^174^ Another well‐described ALS gene modifier is *CX3CR1*.^175^ Deletion of *Cx3cr1* in Tg‐*SOD1^G93A^* mice reduced lifespan, increased neuronal loss and SOD1 aggregation.^176^

Another ALS gene modifier with potential pharmacological application is the *IL6R* C allele that results in the substitution D358A in the interleukin 6 receptor. *IL6R C* ALS carriers have increased levels of IL6 and soluble IL6R in serum and CSF and accelerated disease progression rates;^177^ nevertheless, deletion of *Il6* in Tg‐*SOD1^G93A^* did not alter the lifespan.^178^ Administration of tocilizumab, a humanised monoclonal antibody against IL6R, in SALS patients displaying strong expression of inflammatory genes in peripheral blood mononuclear cells (PBMCs), attenuated clinical symptoms. In contrast, administration of tocilizumab in SALS patients with weak inflammatory gene expression upregulated the inflammatory reaction.^179^ Whether anti‐IL6R therapies may be more effective in *IL6R C* carriers with no *SOD1* mutations remains to be investigated.

## 4 ANTIRETROVIRALS THERAPY FOR ALS

In the 1990s, antibodies against foamy viruses were detected in serum of SALS patients^180^ and a clinical study was initiated to treat SALS patients positive for antibodies against the foamy virus human spuma retrovirus (HSRV), with zidovudine, though it showed no clinical benefit.^181^ Later, the implication of HSRV in ALS patients was challenged^182^ and currently foamy viruses are not considered to participate in ALS.^180^

**FIGURE 7:**
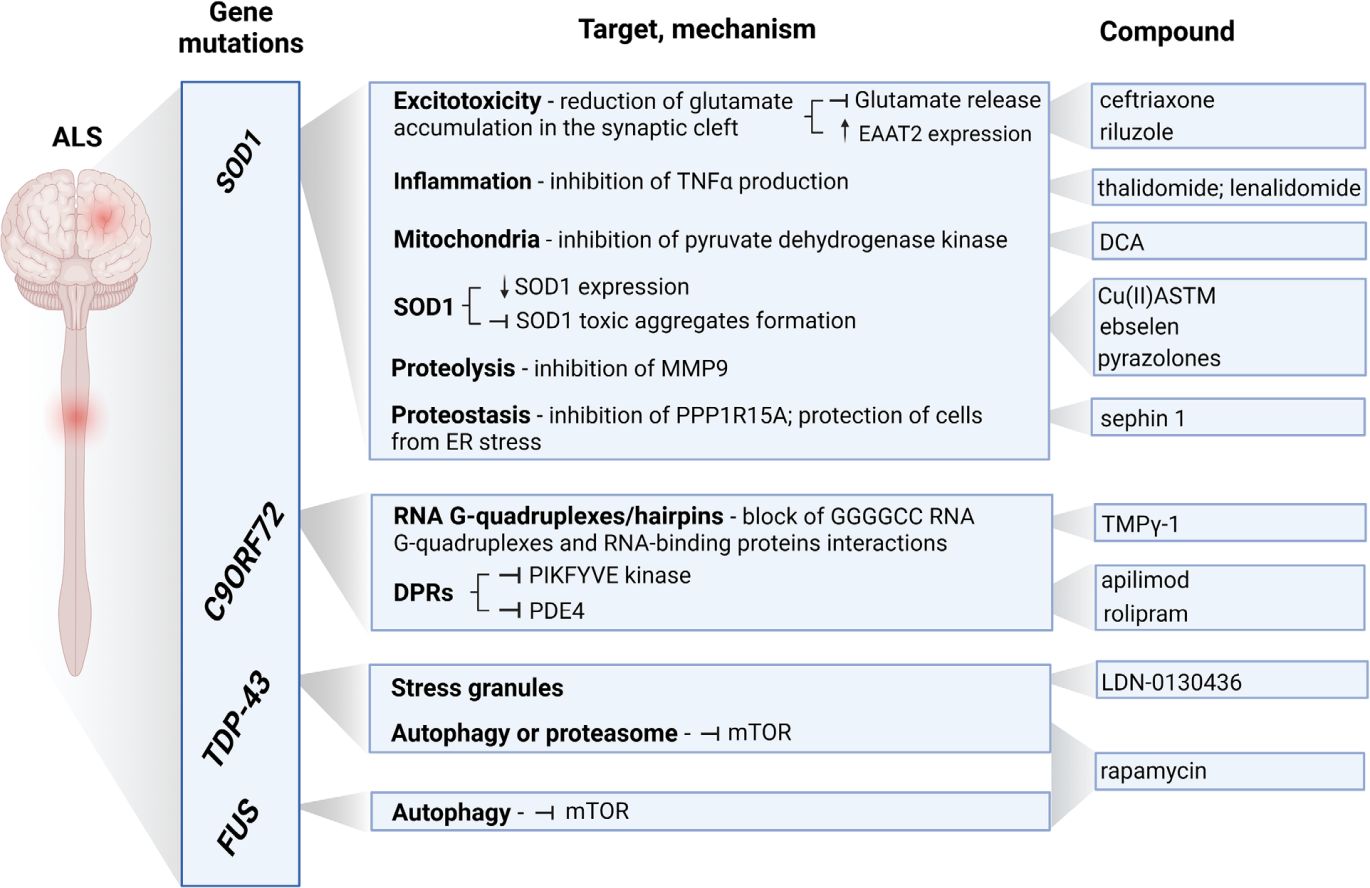
Genotype‐based classification of described ALS subtypes and representative targets and compounds for corresponding patient subtypes. The image was created with Biorender (http://biorender.com)

Human endogenous retroviruses (HERV) represent approximately 8% of the human genome. They are divided into three classes, I, II and III, based on tRNA‐primer binding site. HERV‐K elements belong to class II carrying a complete ORF for *gag*, *pol* and *env* and can produce virus‐like particles.^183^ Expression of HERV‐K was found in a subgroup of ALS patients.^184^, ^185^ TDP‐43 regulates the expression of HERV‐K through binding to the long terminal repeats of the retrovirus.^186^ The *env* viral protein is probably responsible for ALS symptoms since transgenic mice expressing the *env* gene under a neuronal specific promoter show loss of upper and lower motor neurons.^186^ Approved anti‐HIV reverse transcriptase inhibitors block the replication of HERV‐K viral particles by inhibiting the HERV‐K reverse transcriptase,^187^, ^188^ while the integrase inhibitor raltegravir could also block HERV‐K viral particle replication.^188^ Based on these data, in terms of the ongoing clinical trial (NCT02437110), ALS patients with elevated (> 1000 copies/ml) HERV‐K levels are treated with a combination of four anti‐HIV drugs, darunavir, ritonavir, dolutegravir and tenofovir alafenamide. Thus, this study has been designed based on molecular analysis of ALS patients and its completion will define the role of endogenous retroviruses in ALS. The presented ALS subgroups and the compounds that can be directed for their specific treatment are summarised in Figure 7 and Table 3.

**TABLE 3:** Therapeutic approaches for ALS

**Drug name**	**Mechanism of action**	**Preclinical model**	**Effect**	**Replicated in humans**	**Limitations**	**Ref/clinical trial**
**Riluzole**	Anti‐excitotoxic, glutamate release inhibitor	Tg‐*SOD1^G93A^* Tg‐*TDP43A315T* Tg‐*FUS^(1‐359)^* mice	No statistically significant effect in lifespan or motor function	Lifespan extension by 2–3 months	Limited efficacy	^30^, ^31^, ^32^
				No effect in disease progression	Palliative use	
**Ceftriaxone**	Anti‐excitotoxic, increases EAAT2 expression	Tg‐*SOD1^G93A^* mice	Improvement of ALS phenotype	No effect in non‐stratified by genotype ALS patients	Potential mutation dependent therapeutic effect	^33^, ^34^
			Lifespan extension by 10 days	No effect in non‐stratified by genotype ALS patients	Efficacy potentially limited to *SOD1*‐ALS patients	NCT00349622
**Thalidomide**	Inhibitors of TNFα production		Improvement of motor function and body weight			^37^, ^38^, ^39^
**Lenalidomide**			Lifespan extension by 3 weeks			
**Dichloroacetate**	Pyruvate dehydrogenase kinase inhibitor		Improvement of grip strength			^44^
			Lifespan extension by 2 weeks in male and 10 days in female mice			
**Olesoxime**	Mitochondrial pore modulator		Neuronal cell death reduction	No effect in non‐stratified by genotype ALS patients		^45^, ^46^
***Coenzyme Q10***	Antioxidant		Lifespan extension	No effect in non‐stratified ALS patients		^48^, ^49^ NTC00243932
**Vitamin E**	Antioxidant		Delay of disease onset	No statistically significant effect		^51^, ^52^
***Rasagiline***	MAO‐B inhibitor, antioxidant and anti‐apoptotic functions		Improvement of running wheel performance	Reduction of oxidative stress	Results should be confirmed	^55^ NCT01879241 NCT01786603
				Potential modifier of disease progression		
				No effect in survival		
**Pyrimethamine**	Suppressor of SOD1 aggregates formation			Reduction of SOD1 in CSF of *SOD1*‐ALS patients	Long‐term clinical studies should be conducted	^64^ NTC01083667
				Advantage: safe and well‐tolerated		
***Ebselen***	Suppressor of SOD1 toxic aggregates formation	Tg‐*SOD1^G93A^* mice	Delay of disease onset			^76^
**Cu(II)ASTM**	Suppressor of SOD1 toxic aggregates formation	Tg‐*SOD1^G93A^* and Tg‐*SOD1^G37R^* mice	Improvement of ALS symptoms	Ongoing clinical trial	No genotype selection for *SOD1*‐ALS patients	^77^, ^78^ NCT02870634
			Lifespan extension			NCT04082832
***Sephin 1***	Selective inhibitor of PPP1R15A	*Tg‐SOD1^G93A^* mice	Improvement of motor function and body weight			^90^
**Arimoclomol**	Heat shock protein inducer	*Tg‐SOD1^G93A^* mice	Delay of symptoms onset Lifespan extension	No important therapeutic effect in *SOD1*‐mutant ALS		^96^, ^97^, ^98^ NCT00706147
			Improvement of muscle function	Advantage: safe and well‐tolerated		
			Prevention of SOD1 aggregation			
**DB1246 (XX)**	Exhibit high‐affinity binding on GGGGCC RNA G‐quadruplexes	Human iPSC‐motor and iPSC‐cortical neuron cell lines	Reduction of RNA foci and decrease of DPRs		Not validated in ALS patients	^109^
**DB1247 (XXI)**		GGGGCC repeat‐expressing *Drosophilas*	Decrease of DPRs			
**DB1273 (XXII)**			Improvement of survival of larvae			
**Apilimod**	PIKFYVE inhibitor	C9‐BAC mice	Reduction of DPRs			^114^
**Rolipram**	PDE4 inhibitor	Primary hippocampal neurons	Decreased poly(GA)			^116^
**Rapamycin**	Autophagy activator	*Drosophila* ALS/FTD model	Lifespan extension		Different ALS subtypes	^136^
		Tg‐*SOD1^G93A^* mice	Decrease of survival			^137^
**Nilotinib**	Tyrosine kinase inhibitor	Tg‐*TDP‐43^wt^* mice	Neuroprotective effect			^140^
**Bosutinib**		Tg‐*TDP‐43^wt^* mice	Neuroprotective effect			
		iPSC‐derived motor neurons from *TDP‐43*‐ALS or *C90RF72*‐ALS	Increase survival			^141^
		Tg‐*SOD1^G93A^* mice	Delay of disease onset and increase survival			
**Pioglitazone**	PPARγ agonist	Tg‐*TDP‐43^wt^* and Tg‐*TDP‐43^G298S^ Drosophilas*	Improvement of the locomotor function	No effect in combination with riluzole	*Drosophila* models do not recapitulate the corresponding human ALS subtypes or therapeutic effect is mutation dependent	^148^, ^149^ NCT00690118
**Betamethasone**	Antioxidant			Genotype selection for *FUS*‐ALS patients		NTC03707795

## 5 DESIGN OF NEW CLINICAL TRIALS

The genetic background of ALS patients has not been taken into consideration when designing clinical trials, except of those trials which involved oligonucleotide drugs that by default target mutated genes; thus DNA analysis is indispensably required for patient selection. Specifically, the clinical trial NCT02623688 with the antisense drug targeting SOD1 tofersen (BIIB067) enrols only patients with confirmed SOD1 mutations, while the NCT03626012 that involves the BIIB078 targeting *C9ORF72* will enrol only *C9ORF72*‐ALS patients. However, few recent clinical trials with synthetic drug compounds have been designed to enrol ALS patients of a specific genetic background. Arimoclomol (**XXI**) was found to improve muscle strength and prolong survival of Tg‐*SOD1^G93A^* mice by increasing the expression of heat shock protein 70.^96^, ^189^ The clinical trial NCT00706147 with arimoclomol included only patients with confirmed *SOD1* mutations. Arimoclomol was safe and well‐tolerated and further studies are needed to evaluate the therapeutic benefit.^98^ In the same context, a clinical trial with pyrimethamine (**XLIV**) for FALS with *SOD1* mutations to determine the safety and tolerability was performed (NCT01083667). Further, a metformin (**XLV**) clinical trial for treatment of *C9ORF72* ALS patients (NCT04220021) is now recruiting patients. This was based on the fact that although metformin has no beneficial effect in the phenotype of Tg‐*SOD1^G93A^* mice,^190^ in C9‐ALS/FTD mice it mitigated disease symptoms.^191^

### 5.1 Colchicine – an example of genetic analysis to exclude FALS patients

Colchicine is an antiinflammatory drug that can also induce the expression of heat shock protein B8 (HSPB8) that enhances autophagy to remove TDP‐43 of SOD1 misfolded proteins or *C9ORF72*‐related aggregated poly‐dipeptides.^192^, ^193^ An ongoing clinical trial for SALS patients (NCT03693781) excludes patients with mutations in *SOD1*, *TDP‐43*, *FUS* and *C9ORF72*.^192^

### 5.2 Retrospective analysis of outcomes of clinical trials

These studies could reveal relations between chemical treatment and genetic background. In some new clinical trials, ALS patients are screened for certain mutations by DNA sequencing. In certain clinical trials, patients have provided blood samples for future DNA analysis that could be exploited in retrospective studies. Creatine is a representative example. Initially creatine administration to ALS patients did not show any improvement of clinical symptoms.^194^ However, post hoc analysis of clinical data and genetic background of patients showed that ALS patients with A/A and A/C polymorphisms in *MOBP* gene will benefit for creatine treatment.^195^ In the same direction, a meta‐analysis of clinical trials that used lithium carbonate in ALS showed that lithium carbonate increased the 12‐month survival probability from 40% to 70% in patients that carry the C/C polymorphism in *UNC13A*, while no effect in *C9ORF72* carriers.^174^ The basis for this effect is unknown but it appears that pathways involving UCN13A are also regulated by lithium.^196^

### 5.3 Drug combinations

Targeting of multiple ALS pathways can potentially be achieved by administration of drug combinations. In this direction, a combination of ciprofloxacin and celecoxib resulted in significant improvement of motor activity as assessed by swimming distance and velocity in Tg‐*SOD1^G93R^* zebrafish and in zebrafish generated after injection of an mRNA encoding for TDP‐43^G348C^ in one‐cell zygote.^197^ A clinical trial to assess the efficacy of this drug combination in ALS has been initiated (NCT04090684). It is expected that the therapeutic efficacy of drug cocktails administered in stratified patient groups may further improve efficacy.

## 6 TARGET IDENTIFICATION USING GENETICALLY ENGINEERED MODELS AND iPSCs

Genetic ablation or transgenic studies on the Tg‐*SOD1^G93A^* background may unravel new targets for ALS treatment but may not always be of direct clinical relevance since genetic modification has already taken place at the embryo stage and well‐before disease symptoms appear. Drugs are administered when diagnosis has been made, that for ALS may take up to 12 months after the appearance of symptoms. In this sense, a marginal effect on symptoms or life extension after deletion of a gene in an ALS mouse model could indicate that pharmacological targeting of the gene‐encoded protein will not be effective in patients. Inducible knockout models or inducible transgenic models on an ALS background (e.g. Tg‐*SOD1^G93A^*) will provide a more sophisticated practice to search for relevant pharmacological targets since it will allow genetic modification to take place after the appearance of symptoms. In this direction, it has been demonstrated that reduction of *EPHA4* in adulthood does not affect survival of Tg‐*SOD1^G93A^* mice.^198^ Thus, chemical targeting of EPHA4 after the onset of symptoms may not delay disease progression. Finally, extrapolation of animal data to humans should be made in a very cautious manner since mouse models may not recapitulate human ALS, thus some compounds that were found effective in treating mice may not be beneficial to patients. Nevertheless, this problem may be more pronounced when extrapolating data from more distant drosophila and zebrafish.

Testing of new chemical entities for ALS is now boosted by the use of iPSCs generated from patient cells that can recapitulate in vitro the mechanisms of ALS pathology. Indeed, iPSCs have been generated mainly from fibroblasts isolated from FALS and SALS patients. These cells can differentiate to motor neurons but also oligodendrocytes, astrocytes *etc*. iPSCs can be manipulated genetically to correct the mutated gene and generate isogenic control cell lines. The usefulness of iPSCs in the study of ALS but also their limitations as they are in vitro models, and their relatively high cost were reviewed elsewhere.^199^, ^200^

## 7 CONCLUSION

Current clinical and experimental data cumulatively suggest that it is unlikely that a ‘universal’ ALS drug will be effective in ALS patients. In contrast, different subtypes of ALS patients will require different drug treatment strategies according to the suggested chemogenomic approach. In this direction, the use of animal models for testing drugs against a certain genetic background or the re‐evaluation of already completed animal and/or clinical studies is of great importance. As elaborated here, the extrapolation of preclinical data to patients should be performed cautiously since evolutionary distant models such as drosophila and *C. elegans* may provide false findings regarding the efficacy of a drug, in contrast to mice. It is known that the genome and the networks of functional connectivity are significantly different between the more evolutionary distant animals and these could significantly affect the biological outcome of pharmacological treatment. Other factors should also be taken into consideration when evaluating preclinical data, for example, in many preclinical studies, treatment was initiated before the onset of symptoms, which cannot be applied to ALS patients. Currently, diagnosis of ALS takes almost a year since it relies only on the assessment of the clinical status, the electrophysiological examination and progressive exclusion of other pathologies. Nonetheless, the clinical benefit of riluzole is higher when administered early in the course of the disease.^201^ Thus, early diagnosis will enhance therapeutic efficacy. For this, molecular diagnosis of ALS is urgently needed in clinics.^4^ If early drug treatment is combined with patient subgrouping/stratification, it is expected to further extend the clinical benefits of the tested compounds. To this end, it is noted that next‐generation sequencing is important for patient stratification, since it can screen not only for variants in known ALS genes but also in other ALS related genes, such as gene modifiers or drug metabolism‐related genes like the *CYP1A2*.

Finally, it should be noted that there are potential limitations in stratifying ALS patients for treatment given that most ALS cases are sporadic. For SALS, we already know that genetic testing may identify the presence of pathogenic variants in known ALS‐associated genes. However, there will be a large percentage of SALS patients that will lack such variants consequently, these patients cannot be subgrouped. Given that the disease is rare, it may be difficult to recruit large enough cohorts of stratified ALS patients to derive statistically significant conclusions. Further, ethical issues must be considered when stratifying patients based on personalised genetic background.

## CONFLICT OF INTEREST

The authors do not have conflicts to declare.
